# Genomic epidemiological analysis of county-scale *Yersinia pestis* spread pattern over 50 years in a Southwest Chinese prefecture

**DOI:** 10.1371/journal.pntd.0011527

**Published:** 2023-08-07

**Authors:** Jingliang Qin, Liyuan Shi, Yarong Wu, Jinjiao Kong, Xiuwei Qian, Xianglilan Zhang, Xiujuan Zuo, Hang Fan, Yan Guo, Mengnan Cui, Shanshan Dong, Hongli Tan, Youhong Zhong, Yajun Song, Ruifu Yang, Peng Wang, Yujun Cui

**Affiliations:** 1 State Key Laboratory of Pathogen and Biosecurity, Beijing Institute of Microbiology and Epidemiology, Beijing, China; 2 Yunnan Institute of Endemic Diseases Control and Prevention, Dali, China; Colorado State University, UNITED STATES

## Abstract

Plague, one of the most devastating infectious diseases in human history, is caused by the bacterium *Yersinia pestis*. Since the 1950s, the Dehong Dai–Jingpo Autonomous Prefecture (DH) in Yunnan Province, China, has recorded plague outbreaks that have resulted in 1,153 human cases and 379 deaths. The genetic diversity and transmission characteristics of *Y*. *pestis* strains in this region remain unknown. Here, we performed high-resolution genomic epidemiological analysis of 175 *Y*. *pestis* strains isolated from five counties and 19 towns in DH between 1953 and 2007. Phylogenetic analysis revealed that most DH strains were located in lineage 1.ORI2, which could be further subdivided into seven sub-phylogroups (SPG1-SPG7). The dominant sub-phylogroups of *Y*. *pestis* in DH varied during different periods and presented a population shift. Genomic evidence showed that plague might have emerged from the southwest of DH (e.g., Longchuan or Ruili counties) or its bordering countries, and subsequently spread to the northeast in multiple waves between 1982 and 2007. Our study infers a fine-scale phylogeny and spread pattern of the DH *Y*. *pestis* population, which extends our knowledge regarding its genetic diversity and provides clues for the future prevention and control of plague in this region.

## Author summary

*Yersinia pestis*, the causative agent of plague, has led to three historic pandemics and remains a major public health issue. Here, genomic epidemiological analysis of 175 *Y*. *pestis* strains isolated over 50 years from the Dehong Dai—Jingpo Autonomous Prefecture, a region located in southwestern China, revealed the genomic evolution scenario of *Y*. *pestis* with high resolution. Our results revealed that plague epidemics in this region were led by different phylogroups, and population shift events were observed during different years, suggesting high dynamics of *Y*. *pestis* populations in the local natural plague foci. We found that the spread of plague was from southwest to northeast and inferred the possible origin of the epidemics, which would help strengthen surveillance of the epizootic in local natural plague foci and promote the prevention and control of human plague.

## Introduction

Plague is a deadly infectious disease caused by *Yersinia pestis* that can spread to humans through infected rats, fleas, or airborne droplets under certain conditions [1–3]. There are three main clinical forms of plague infection: bubonic, pneumonic, and septicemic [4]. Considering the widespread natural foci around the globe, the high fatality rate, and the ability to spread through the respiratory tract, plague remains a major public health issue worldwide [5–9].

Dehong Dai—Jingpo Autonomous Prefecture (DH), a prefecture of Yunnan, China, shares more than 500 km of land border with Myanmar and is an important trade center linking China and Southeast Asia [10]. The natural plague foci in northern Myanmar near the China-Myanmar border have a similar climate, vegetation landscape, and animal and insect distribution to those in DH, China. The similar ecological environment, together with frequent cross-border trade and personnel flow provide opportunities for plague to spread between DH, Myanmar, and other neighboring countries. The documented plague outbreak in DH could be dated back to 1938 [11], with *Rattus tanezumi* (*R*. *tanezumi*) as the major rodent host. Between 1938 and 1949, a total of 9,134 confirmed infections and 4,259 deaths were reported, with a fatality rate of 46.63%. Since the 1950s, the Yunnan Institute of Endemic Disease Control and Prevention has been established, and active animal plague surveillance works have been conducted annually [12]. Although human plague epidemics have declined nowadays, plague remains endemic in South Yunnan [13–15].

Genetic diversity surveys could help understand the genetic characteristics and spread patterns of *Y*. *pestis*. Multiple molecular genotyping methods, including multiple loci VNTR, different regions, CRISPR, and pulsed-field gel electrophoresis analyses [16–21], have been used to investigate the genetic diversity of *Y*. *pestis* strains isolated in DH, which facilitates their rapid screening and preliminary population assignment. However, the low phylogenetic resolution of these methods limits the inference of fine-scale phylogeny and transmission details of strains isolated in this region. Genomic epidemiological studies based on genome-wide single-nucleotide polymorphisms (SNPs) have proven effective in rebuilding high-resolution evolutionary histories at both global and local scales, providing evidence for the spread of both recent outbreaks and historical epidemics [6,8,22].

To investigate the genetic diversity and spread pattern of *Y*. *pestis*, we collected and sequenced the whole genomes of 175 *Y*. *pestis* strains isolated over 50 years in the DH *R*. *tanezumi* plague focus. By combining whole genome-wide diversity analysis and local epidemiological documents, we reconstructed the phylogenetic tree of DH *Y*. *pestis* strains and inferred the possible driving forces related to their spread.

## Methods

### *Y*. *pestis* strain collection and DNA extraction

In this study, 175 *Y*. *pestis* strains were collected from DH *R*. *tanezumi* plague focus between 1953 and 2007 by the local centre for disease control and prevention, as well as Yunnan Institute of Endemic Diseases Control and Prevention during routine surveillance and plague outbreaks (S1 Table). Mouse traps and cages were placed near host animal burrows and residential area, respectively, and monitored for three consecutive days to identify live rodents in the vicinity of the surveillance area. Concurrently, unexplained dead rodents were discovered during epidemiological investigations, following the standard protocol of the National Scheme of Plague Surveillance of China (http://www.gov.cn/yjgl/2005-08/30/content_28245.htm). Additionally, individuals presenting with high fever, severe illness, and/or having contact history with confirmed plague cases or exposure to the plague were also examined.

Samples, including lymph fluid from patients, liver and spleen from captured live rodents, gastric contents of fleas on captured rodents, heart, gland, lung, spleen, liver, bone marrow from dead rodents, and soil samples, were cultured on plates [23]. *Y*. *pestis* strains were isolated and identified from these samples in accordance with Appendix B of the Diagnostic criteria of plague, WS279-2008, within the Health of the People’s Republic of China industry standards.

*Y*. *pestis* strains were preserved using fresh degreased pure milk, then lyophilized and stored at -20°C. When DNA extraction was required, the preserved strain was inoculated into LB medium with added blood and cultured at 28°C for 24 hours. After three passages, DNA was extracted using a QIAGEN DNeasy Blood & Tissue kit (QIAGEN Shanghai, China, No. 69506). Gel electrophoresis was conducted for quality control prior to next-generation sequencing [23].

### Whole Genome sequencing and assembly of *Y*. *pestis* strains

A total of 514 *Y*. *pestis* genomes were used in this study, including 175 newly sequenced genomes collected from the DH *R*. *tanezumi* plague focus (S1 Table) and 339 global isolates downloaded from NCBI (up to August 2018, S2 Table). Among the 175 genomes, three were isolated from Myanmar near the China-Myanmar border, and the remaining 172 were collected from different municipal regions of DH.

Genomic DNA libraries of the 175 *Y*. *pestis* strains were prepared using a TruSeq DNA Library Preparation Kit following the manufacture’s recommendation and sequenced on an Illumina X-Ten sequencing platform with a 150 bp paired-end sequencing library. The sequencing quality was assessed using FastQC (v0.11, https://www.bioinformatics.babraham.ac.uk/projects/fastqc/) to ensure high-quality data for all samples. Raw reads were filtered using Trimmomatic (v0.40) [24], removing low-quality reads with a mean Phred quality score below 20. The filtered reads were then assembled using the SPAdes (v3.11) software [25].

### SNP calling

The assemblies were aligned against the chromosome of the reference *Y*. *pestis* CO92 genome (NC_003143.1) using MUMmer (v3.0) [26] to identify SNPs in the core genome. The SNPs located in repetitive regions were removed. The identified SNPs based on assemblies were further verified by mapping the sequencing reads to the reference using BWA (v0.7) [27] and GATK (v3.8) [28,29]. High-quality SNPs in each strain required Phred base quality scores > 20, at least 10 supporting reads, and 90% allele frequency. Finally, high-quality SNPs present in at least 95% of all genomes were retained for further analysis.

### Phylogenetic analysis

A total number of 3,483 high-quality SNPs were identified in a dataset of 514 strains. Then, concatenated SNPs were used to construct a maximum likelihood (ML) tree with IQ-TREE (v1.6) [30] under the Generalized Time Reversible model with a bootstrap value of 100.

To obtain high-resolution topology for DH strains, we recalled the SNPs for 172 of 175 newly sequenced genomes located in phylogroup 1.ORI2 using the same pipeline. In total, 132 high-quality SNPs were identified in the dataset of 172 DH strains, with CO92 acting as the outgroup. Bionumerics 6.6 software (Applied Maths NV, 2012) and IQ-TREE (v1.6) [30] were used to construct a minimal spanning tree and an ML tree for DH strains in a 1.ORI2, respectively.

### Spread pattern inference

The spread of *Y*. *pestis* strains between different municipal regions within DH was assessed based on 172 DH strains located in phylogroup 1.ORI2 using the “evobiR” (v1.1) and “phytools” (v0.7–80) packages for R [31,32]. Source records (municipal regions) were matched to the tips of the ML tree using the ReorderData function in EvobiR. Stochastic source mapping was then performed under the ARD model with 100 replicates using the “make.simmap” function in Phytools.

The numerical data used in all figures are included in S1 Data.

## Results

### *Y*. *pestis* sampling in DH

**Fig 1:**
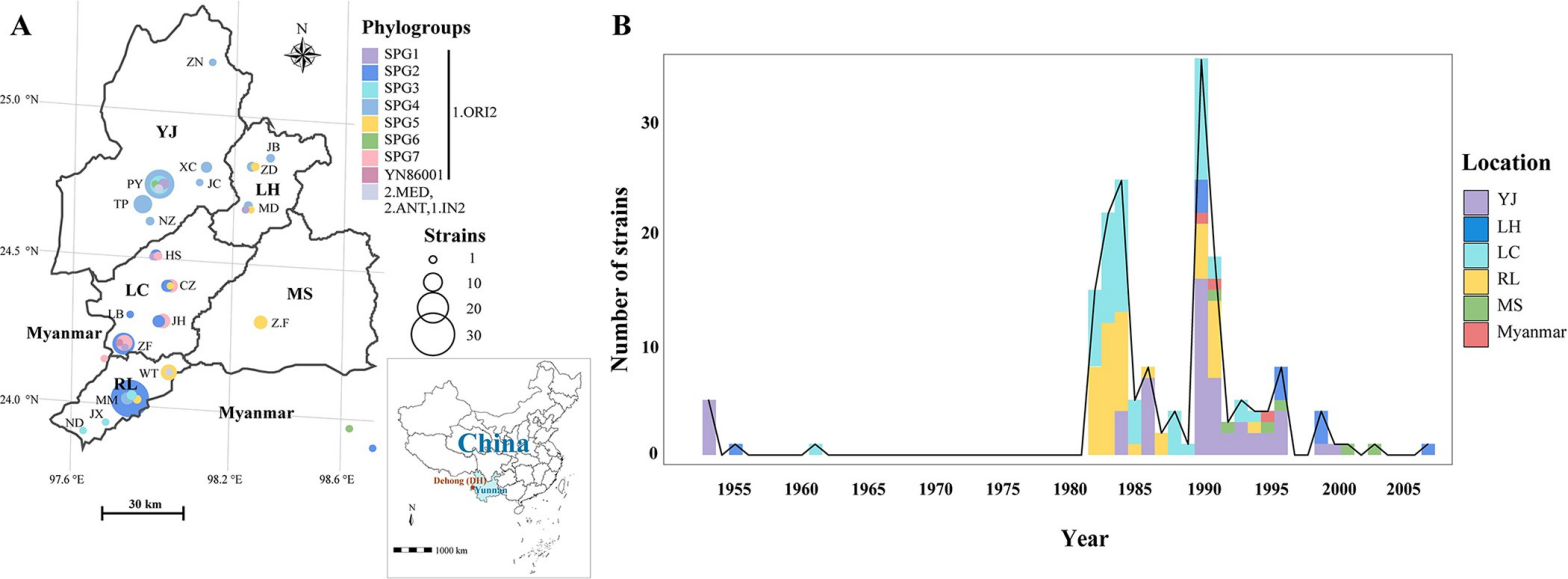
Distribution of *Y*. *pestis* strains in the DH *Rattus tanezumi* plague focus between 1953 and 2007. (A) Spatial and (B) temporal distribution of 175 newly sequenced strains. Geographic abbreviations: Yingjiang (YJ), Lianghe (LH), Longchuan (LC), Ruili (RL), and Mangshi (MS). Smaller letters indicate towns (for example, ZN) Town abbreviations: Zhina (ZN), Xincheng (XC), Jiucheng (JC), Nongzhang (NZ), Pingyuan (PY), Taiping (TP), Husa (HS), Chengzi (CZ), Jinghan (JH), Zhangfeng (ZF), Longba (LB), Wanding (WT), Mengmao (MM), Jiexiang (JX), Nongdao (ND), Zhefang (Z.F), Jiubao (JB), Zhedao (ZD), Mangdong (MD). Circle size represents the number of strains. Different colors represent phylogroups or counties, as shown in the legend. The map was created using the “ggplot2” (v3.4.1) and “sf” (v1.0–13) packages for R software (v4.2.2). The base layer used in the map was obtained from Amap’s open data platform (http://datav.aliyun.com/portal/school/atlas/area_selector).

Active plague surveillance programs in DH have been conducted annually since the 1950s. From 1953 until now, plague has been endemic in five DH counties, including 1,153 human cases and 379 deaths [33–35]. A total of 175 *Y*. *pesti*s strains from the DH *R*. *tanezumi* plague focus were collected during 1953–2007, including 172 strains sampled from five counties and 19 towns in DH and three strains from Myanmar (Fig 1A and S1 Table). Most of the strains were isolated after 1982 (168/175, 96.00%), and seven strains were isolated previously (Fig 1B and S1 Table).

### Genetic diversity of *Y*. *pestis* in DH

**Fig 2:**
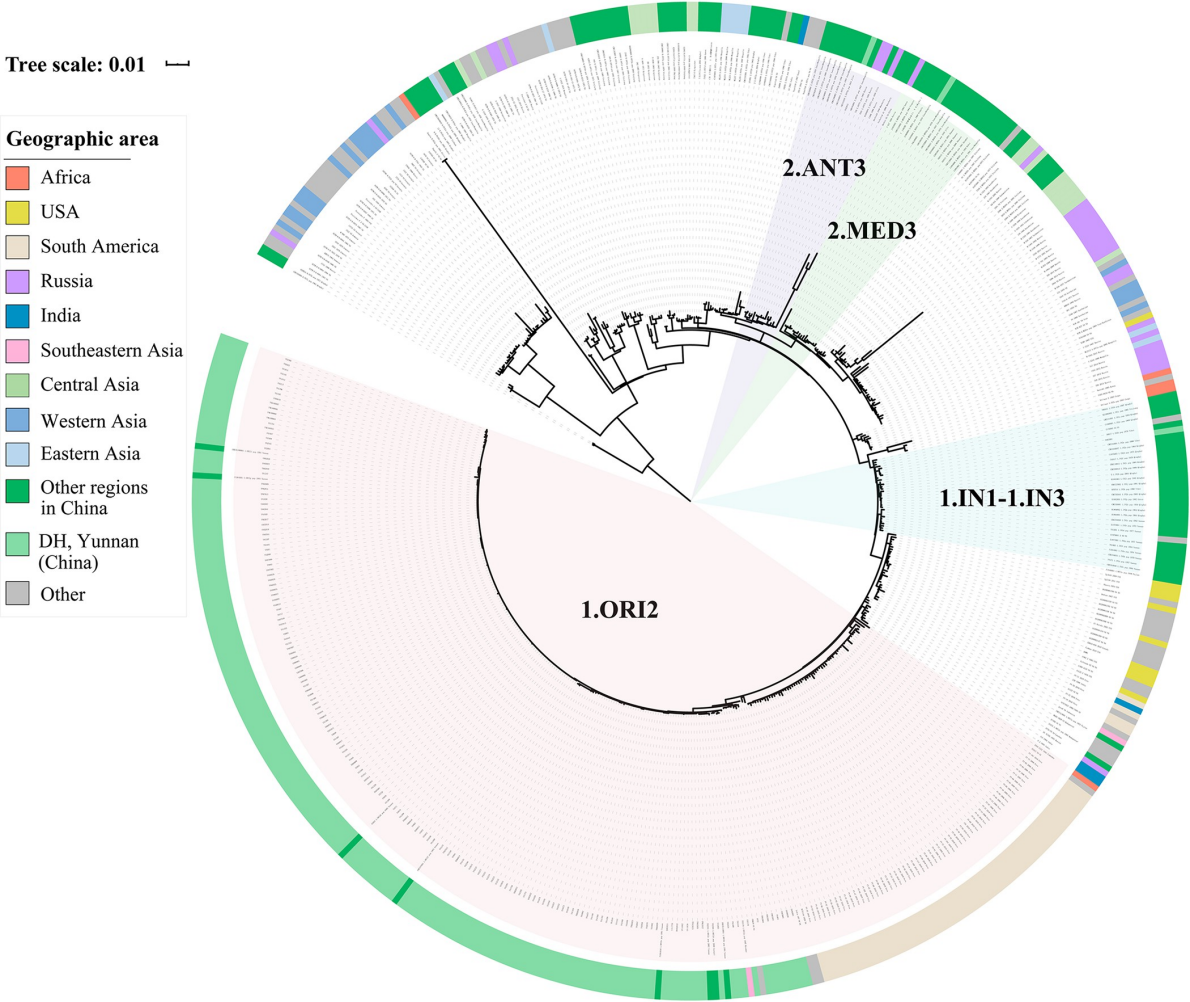
Phylogenetic positioning of the Dehong Dai-Jingpo (DH) *Y*. *pestis* strains. A maximum likelihood phylogeny including 175 DH strains and 339 publicly available genomes was generated based on 3,483 SNPs in the core genome. Isolation location for each strain is indicated in the outer circle. For clarity, only phylogroups involving DH strains are marked.

To characterize the phylogenetic positions of the DH isolates in the genealogy of *Y*. *pestis*, we compared the 175 newly sequenced strains with 339 published genomes (S2 Table) to reconstruct the phylogeny. In total, 3,483 SNPs were identified across all samples and were used to build the tree, which showed that 98.29% (172/175) of the DH strains belonged to phylogroup 1.ORI2 (Fig 2), whereas only one strain was identified for each of the other three phylogroups, including 1.IN2, 2.MED3, and 2.ANT3. Therefore, 1.ORI2 is the dominant *Y*. *pestis* strain in the DH population.

**Fig 3:**
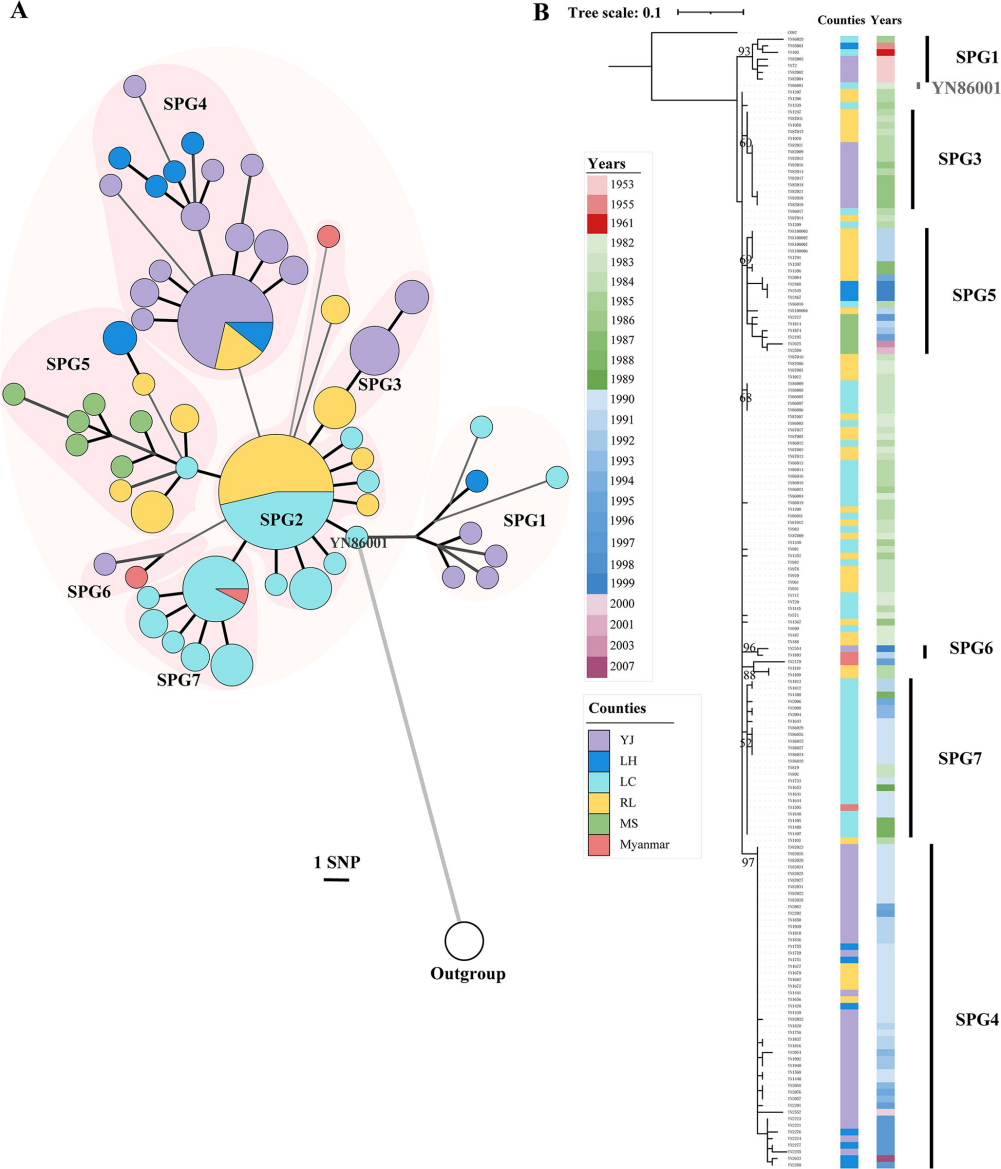
Population structure of DH *Y*. *pestis* strains located in lineage 1.ORI2. (A) A minimal spanning (MS) tree of 172 DH *Y*. *pestis* strains based on 132 SNPs in the core genome, using the *Y*. *pestis* CO92 strain as the outgroup. Pink circles indicate sub-phylogroups. The circle size of the MS tree represents the number of strains, while different colors of the circle represent geographical isolation locations, abbreviated as follows: Yingjiang (YJ), Lianghe (LH), Longchuan (LC), Ruili (RL), and Mangshi (MS). (B) A maximum likelihood tree of 172 DH *Y*. *pestis* strains based on 132 SNPs in the core genome, using the *Y*. *pestis* CO92 strain as the outgroup. Sub-phylogroups are labeled using black lines; SPG2 strains located near the root of the phylogeny are unmarked. Isolation dates and locations are shown in different colors.

To further investigate the details of the phylogenetic relationships among DH strains, we reconstructed the phylogeny using the 172 1.ORI2 strains isolated from DH using the *Y*. *pestis* CO92 strain of 1.ORI1 as the outgroup. In total, 132 SNPs were identified. After excluding the unique CO92 SNPs, only 104 SNPs remained for the DH 1.ORI2 strains, with an average pairwise genetic distance of four SNPs between them, suggesting a high clonal characteristic of *Y*. *pestis* in DH. According to the phylogeny, DH strains could be attributed to seven sub-phylogroups, named SPG1-SPG7 (Fig 3A). Notably, one strain (YN86001) was located at the position of the most recent common ancestor (MRCA) of all seven sub-phylogroups (Fig 3A), which provided clues for the original source of the plague epidemics in DH.

### Population shift events in DH

**Fig 4:**
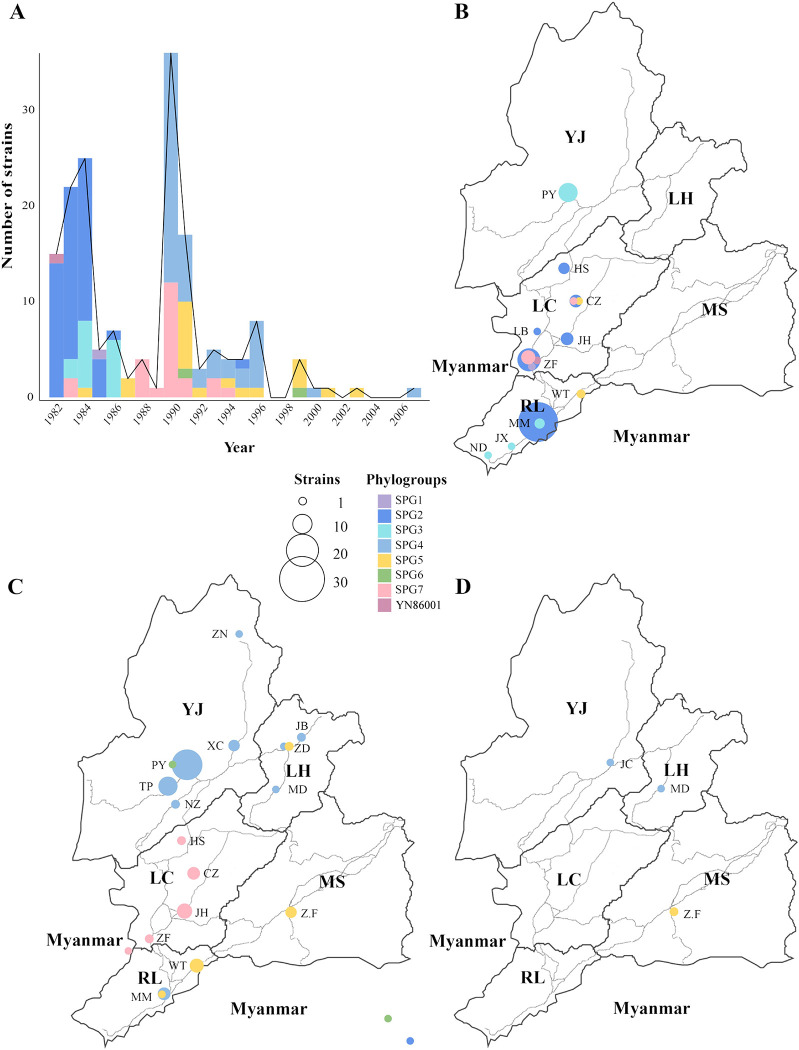
Dynamic changes in *Y*. *pestis* subpopulations in the DH *Rattus tanezumi* plague focus after 1982. (A) Temporal distribution of DH *Y*. *pestis* subpopulations isolated after 1982. (B) Geographical distribution of DH *Y*. *pestis* subpopulations isolated between 1982 and 1989. (C) Geographical distribution of DH *Y*. *pestis* subpopulations isolated between 1990 and 1999. (D) Geographical distribution of DH *Y*. *pestis* subpopulations isolated between 2000 and 2007. The boundaries of urban surfaces are illustrated using black lines. Light grey curves indicate traffic lines. Geographic abbreviations: Yingjiang (YJ), Lianghe (LH), Longchuan (LC), Ruili (RL), and Mangshi (MS). Town abbreviations: Zhina (ZN), Xincheng (XC), Jiucheng (JC), Nongzhang (NZ), Pingyuan (PY), Taiping (TP), Husa (HS), Chengzi (CZ), Jinghan (JH), Zhangfeng (ZF), Longba (LB), Wanding (WT), Mengmao (MM), Jiexiang (JX), Nongdao (ND), Zhefang (Z.F), Jiubao (JB), Zhedao (ZD), Mangdong (MD). The map was created using the “ggplot2” (v3.4.1) and “sf” (v1.0–13) packages for R software (v4.2.2). The base layer used in the map was obtained from Amap’s open data platform (http://datav.aliyun.com/portal/school/atlas/area_selector).

We noticed that the dominant subpopulations of *Y*. *pestis* in DH varied during different periods. Before 1982, all six strains isolated in DH belonged to the SPG1 group (Fig 3B). Focusing on strains isolated after 1982, we found that SPG2 was the first dominant population during this period, which mainly circled multiple municipal counties in Longchuan (LC), Ruili (RL), accounting for 72.97% (54/74) of the total strains isolated between 1982 and 1986 (Fig 4A). Between 1988 and 1996, a co-epidemic of SPG4 (47/82, 57.32%) and SPG7 (22/82, 26.83%) replaced SPG2 as the major population, which was predominantly distributed in RL, LC, Lianghe (LH), and Yingjiang (YJ). The other sub-phylogroups, including SPG3, SPG5, and SPG6, also appeared after 1982, but were scattered as minor populations in the region.

### Local spread of *Y*. *pestis* in DH

Most DH strains belonged to phylogroup 1.ORI2 and were isolated after 1982 (166/175; 94.86%). We investigated the cross-county spread of DH 1.ORI2 strains during the epidemics. Most strains isolated from RL or LC in the 1980s (between 1982 and 1989) were located on the backbone of the phylogenetic branch (SPG2), with few or no SNP differences, which represented a clonal outbreak of *Y*. *pestis* during this stage (Figs 3B, S1 and S3 Table). We also found that YN86001, located precisely at the position of the MRCA of SPG1-SPG7, was also isolated from LC on July 28, 1982 (Fig 3B). These results imply that the plague might originate from LC or RL (located in the southwest of DH) or its bordering countries. *Y*. *pestis* spread was oriented RL to YJ (southwest to north of DH) between 1982 and 1989 (Figs 4B and S1).

**Fig 5:**
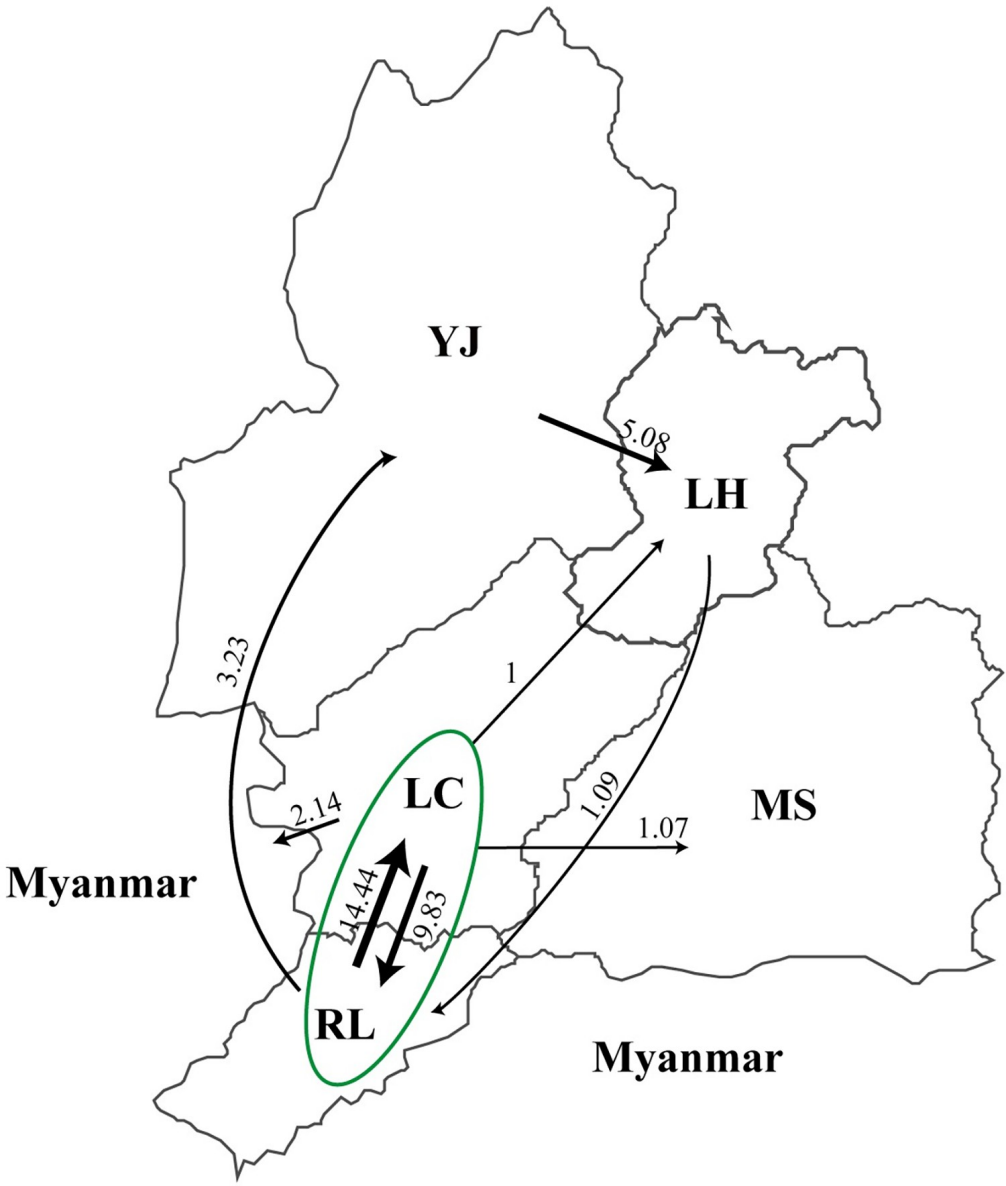
Southwest to northeast spread routes of *Y*. *pestis* isolated after 1982. Black lines represent the spread routes, and arrows indicate the direction of spread. Numbers indicate the spread events. Counties within the green circle indicate the possible source of DH plague epidemics. Geographic abbreviations: Yingjiang (YJ), Lianghe (LH), Longchuan (LC), Ruili (RL), and Mangshi (MS). The map was created using the “ggplot2” (v3.4.1) and “sf” (v1.0–13) packages for R software (v4.2.2). The base layer used in the map was obtained from Amap’s open data platform (http://datav.aliyun.com/portal/school/atlas/area_selector).

After 1989, more spread events from the southwest of DH to the north and northeast were observed, with two newly involved regions, LH and Mangshi (MS) (Fig 4C). Plague was first introduced into LH and MS in 1990 and 1991, respectively (Fig 3B). Phylogenetic analysis revealed that *Y*. *pestis* isolated in MS (SPG5) spread from LC (67% possibility) or RL (40% possibility) through one spread event, whereas plague was introduced to LH through at least six individual spread events: one from LC or RL (SPG5) and five from YJ (SPG4) (Figs 5, S1 and S4 Table). After 2000, the intensity of plague epidemics decreased, with only four strains isolated from the northeast, and no strains from the west or central region of DH (Fig 4D).

## Discussion

Previous studies have revealed that DH has the longest period of plague epidemics and the largest area of natural plague foci within the Yunnan Province [15,33,34]. The abundant strains isolated from the local region provided us with an opportunity to investigate the cross-county spread pattern of *Y*. *pestis* in DH. Our results showed that the plague might have originated from LC, RL, or bordering countries, and then spread from the southwest of DH to the northeast. Interestingly, we found that one strain, YN86001, located at the position of the MRCA of SPG1-SPG7 in the phylogeny (Fig 3A). As YN86001 was isolated from LC in 1982, LC might be the key region that caused plague epidemics in DH. In addition, genomic evidence showed that *Y*. *pestis* strains isolated in Myanmar and the northeast of DH were likely introduced from LC and RL through several independent spread events, and that there were frequent interchanges between LC and RL (Fig 5). According to a 2011 report, up to 15.2 million people passed through the frontier inspection station in RL, an important trade center linking China, South Asia, and Southeast Asia [36,37]. By 2019, this number increased to 17 million [38]. In addition, between 2005 and 2017, a total of 40 events and 8,252 cases of other infectious disease from Myanmar, Laos and Vietnam were reported in Yunnan Province, with the number of imported infectious diseases increasing annually [39]. Though we do not have direct data on the spread of plague due to transportation and population movement, given the established role of these factors in the spread of other infectious diseases in Yunnan, it is reasonable to suggest that similar risks could apply to the spread of the plague within DH or between DH and neighboring countries. Moreover, the administrative center of LC is only 29.1 km from RL, and the climate of the two counties is similar, with relatively constant temperature and humidity suitable for rodent survival. There are no natural barriers, such as high mountains, between LC and RL, which may facilitate *Y*. *pestis* spread.

Notably, we observed population shift events in DH *Y*. *pestis* strains, with dominant subpopulations varying across different periods (Figs 3B and 4). As previously reported, the diversity and abundance of rodent reservoirs, ecological changes, random population fluctuations, and the reintroduction of new strains from other regions could drive population shifts [6,40–43]. In addition, active interventions, such as elimination of rodents and fleas and vaccination against *Y*. *pestis*, can also act as important driving forces [44,45]. The end of the DH *R*. *tanezumi* plague epidemic in the late 1950s, accompanied by the decline of SPG1, was closely associated with large-scale anti-plague interventions [46]. Subsequently, plague in DH remained silent for more than 20 years, with only a few attenuated *Y*. *pestis* strains and bacteriophages observed in the local natural plague foci [15]. Similar human interventions were implemented following each outbreak, leading to a temporary vacuum of *Y*. *pestis* and low host/vector density in the niche of local natural plague foci. With the recovery of the host and vector populations, *Y*. *pestis* from adjacent regions or the minor latent population surviving in the soil or rodent reservoirs, might emerge and lead to a new round of plague epidemics, which appeared as a shift in *Y*. *pestis* populations. For example, SPG4, replacing SPG2 and SPG3, became the dominant subpopulation in YJ in the 1990s since the introduction of *Y*. *pestis*, probably from LC, RL, or bordering countries (Fig 4A–4C). The migration of host animals and human activities, such as boarding trade and public transport, might promote the introduction and establishment of new populations.

Several limitations exist in the current study, mainly including sampling bias. First, there was a shortage of *Y*. *pestis* strains isolated in DH before the 1980s, with only seven strains included in this study owing to insufficient manpower and resources in the 1950s and the silence of local plague foci during the 1960s and the 1970s [34,46,47]. Thus, it is difficult to infer the genetic diversity and spread pattern of *Y*. *pestis* strains during this period and compare them with epidemic strains after the 1980s. Second, *Y*. *pestis* strains were occasionally collected from neighboring Yunnan and other Southeast Asian countries. Only three Myanmar strains were collected after 1982. Thus, there was insufficient genomic evidence to infer the cross-border spread of *Y*. *pestis*.

In this study, we analyzed the genetic diversity and county-scale spread pattern of *Y*. *pestis* strains isolated over 50 years in DH along the Myanmar border. Phylogroup 1.ORI2 formed the dominant population of DH strains, which could be further subdivided into seven sub-phylogroups, with population shift events occurring during different periods. Importantly, we found that the plague in DH might have originated from the southwest (e.g., LC or RL counties) or its bordering countries, and then spread to the northeast. DH is an import gateway connecting China and Southeast Asia, where natural plague foci are widely distributed and plague epidemics are particularly persistent in Yunnan. Thus, illuminating the fine-scale phylogeny and county-scale spread pattern of DH strains will help the prevention and control of both domestic plague and cross-border transmission events.

## Supporting information

S1 Fig Accessed transmission of the DH *Y*. *pestis* strains among different counties after 1982 based on the Bayesian Markov Chain Monte Carlo method. (TIF)

S1 Table Background information of the 175 *Y*. *pestis* strains in DH Prefecture, Yunnan. (XLS)

S2 Table Background information of the 339 published genomes used in this study. (XLSX)

S3 Table Distribution of *Y*. *pestis* populations in different DH counties. (XLSX)

S4 Table Plague spread events in DH after 1982. (XLSX)

S1 Data Spreadsheets containing tables with the numerical data used for Figs 1B, 4A, 5 and S1. (XLSX)
