# Supplementary figures and images for: Genomic epidemiological analysis of county-scale Yersinia pestis spread pattern over 50 years in a Southwest Chinese prefecture
Source: PLoS Negl Trop Dis. 2023 Aug 7;17(8):e0011527. doi: 10.1371/journal.pntd.0011527 (PMC10406180; doi:10.1371/journal.pntd.0011527)

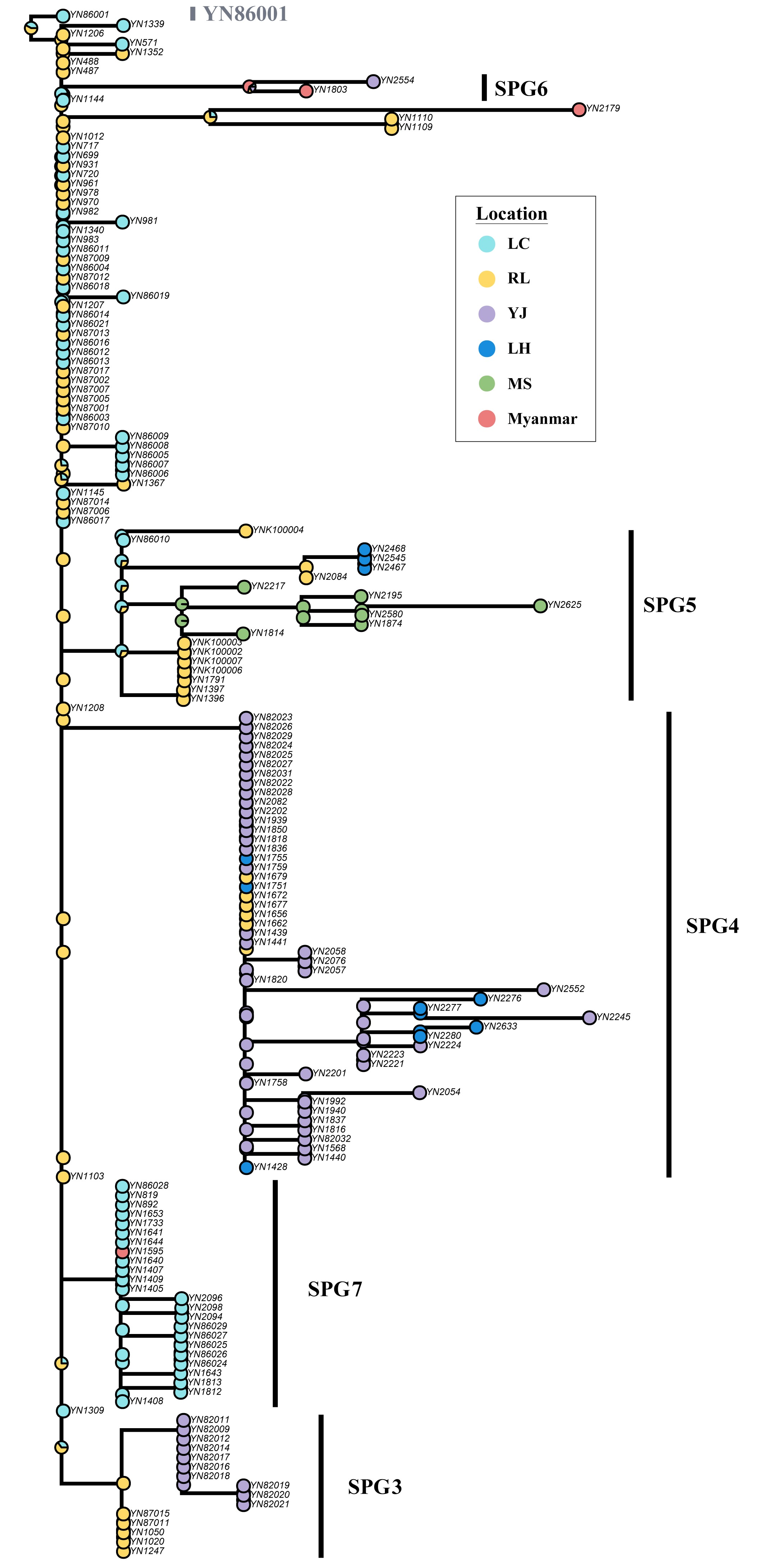

Supplement: S1 Fig — (TIF) [file pntd.0011527.s001.tif]
